# “You must cut that long and stinking thing”: uncovering the lived experiences of uncircumcised pokot women in North-Eastern Uganda

**DOI:** 10.1186/s12905-022-02005-4

**Published:** 2022-11-04

**Authors:** Noah Kalengo, Laban. K. Musinguzi, Janestic Mwende Twikirize

**Affiliations:** 1 Department of Social Work and Social Administration, School of Social Sciences, College of Humanities and Social Sciences, Makerere University, Kampala, Uganda; 2 Department of Social Work, Faculty of Social Sciences, Kyambogo University, Kampala, Uganda

**Keywords:** Pokot, Uncircumcised women, Lived experiences, Sexual enjoyment, Serial interviewing

## Abstract

**Background:**

Female circumcision remains a dominant practice among the Pokot of North-Eastern Uganda. This paper explores the lived experiences of uncircumcised Pokot women, as they continue to live in a community, where the practice is cherished.

**Methods:**

This qualitative study adopted an ethnographic research design. The study was based on thirty [30] serial interviews with 15 uncircumcised women in the Pokot local language between August and October 2021. Five [5] Key Informant Interviews were also conducted with key informants from Amudat District. A Focus Group Discussion with women, irrespective of their circumcision status, was organized as an entry point to identify the initial uncircumcised woman. Uncircumcised women were recruited using respondent-driven sampling while key informants were purposively selected. Data were analyzed thematically. Participants were allocated codes to ensure anonymity.

**Results:**

Participants expressed understanding of female circumcision, and the procedure although they were not circumcised. Denial of participation in community and cultural functions, rejection by elders and relatives, difficulties in getting marriage partners, denial of conjugal rights and basic needs, refusal to give names to their children, and home desertion were the negative experiences reported by uncircumcised women. Sexual enjoyment during sexual intercourse, epitomized by the ease of reaching orgasms, fewer complications while giving birth as well as reduced risk of exposure to sexually transmitted diseases were mentioned by participants as their positive experiences.

**Conclusion:**

Uncircumcised Pokot women continue to experience unbearable challenges since female circumcision is perceived as the only rite of passage to womanhood. This calls for intensified awareness of the population on the challenges associated with female circumcision refusal while demonstrating the positive experiences mentioned by uncircumcised women, that can be exploited as the beacon of hope.

## Background

The World Health Organization (WHO) [1] defines female circumcision as ‘all actions involving injury to the female genital organs for non-medical reasons. International and regional legal instruments classify female circumcision as a human rights violation, with over 200 million girls and women across the world estimated to have undergone the practice [1–6]. There are contestations between human rights activists and African communities practicing female circumcision regarding the contextual definition of female circumcision [2, 3]. African communities accuse human rights activists of using Female Genital Mutilation (FGM) as a way of making the practice look barbaric, dehumanizing, and a violation of the human rights of girls and women [2, 3]. Female circumcision in countries like Somalia, Mali, Guinea, Djibouti, Sudan, and Egypt is considered an accepted practice [7]. In most of these countries, the prevalence of female circumcision remains at approximately 90% [7–10]. The practicing communities raise many contextual factors to justify the practice including; proper hygiene or cleanliness among women, marriageability, a rite to passage from childhood to womanhood, low sexual desire among the circumcised, wealth accumulation and women’s economic dependency [9–16].

In Uganda, female circumcision is practiced among the Sabiny in Kapchorwa, Bukwo and Kween Districts in Elgon sub-region as well as the Pokot, Tepeth, and Kadam in Nakapiripirit, Moroto and Amudat Districts in Karamoja sub-region [17, 18]. Although the prevalence of female circumcision at the national level has reduced to 1.4%, the practice is still dominant among the Pokot of Amudat District [3]. Evidence indicates that, over 54% of the Pokot girls and women have undergone female circumcision [17, 18]. Fortunately, this has reduced, compared to Uganda Demographic and Health Survey (UDHS) [1], which estimated the prevalence at 95%, and Uganda Women Network at 80% in 2016 [4]. The persistence of female circumcision among the Pokot is associated with strong social values, and high illiteracy levels (42.4%) among women [3]. This has been exacerbated by the psychosocial benefits tagged to the practice such as; social acceptance, marriage blessings from elders, respect from relatives, equal status of belonging and citizenship, passage to womanhood, and social inclusion [4].

Nonetheless, the sustained campaigns against female circumcision by state and non-state actors backed by legal instruments have led to an increasing number of uncircumcised girls and women among the practicing communities [5–13, 21–25]. According to the WHO (2018), annually about 30 million uncircumcised girls and women (15–49 years) in Africa and Asia are at risk of undergoing the practice due to increasing pressure from their relatives and parents. Besides, uncircumcised girls and women are viewed by the practicing communities as deviant people who have gone against the culturally accepted norm [2, 15–18, 26–28].

The refusal by uncircumcised women to bow to the socio-cultural norms that define them in the community alienates them from their communities and thus they cease to have an equal status of belonging as compared to those that are receptive[17]. Ultimately, they lose the entitlements of belonging [11, 29–36]. Culturally accepted norms like female circumcision are backed by societal norms that must be followed by families to be accepted within their communities [11, 12, 37–39]. These social norms pressure parents to persuade their daughters to undergo the practice in order to prepare them for marriage and adulthood [11, 31–34].

Notwithstanding the restrictions against female circumcision, pressure on the uncircumcised women to respect and adhere to the social and cultural values that define them is often rife [10, 23, 44–46]. As a result, uncircumcised women that cannot stand the social pressure from their parents, marital partners, relatives and friends have been forced to secretly undergo female circumcision in their communities [15, 20, 41, 42, 47, 48]. Nonetheless, some of the uncircumcised women have stood their ground against the practice [5, 17, 18, 45, 49]. However, little evidence is available to explain their lived experiences as they continue to live in a community that considers them deviants. It is upon such a backdrop that this study sought to explore the lived experiences of the uncircumcised Pokot women in North-Eastern Uganda.

## Methods

### Research design

We adopted an ethnographic research design guided by interpretive paradigm [42–45]. The adoption of ethnographic research design and interpretative approach was informed by the desire to gain a deeper understanding of the lived experiences of uncircumcised Pokot women from their own perspective. The design allowed us to carry out repetitive visits and engross in the community for effective interaction with the participants.

### Setting and participants

We conducted the study among the Pokot community in North-eastern Uganda, where over 54% of the women are circumcised compared to the national prevalence of 1.4% [17, 18]. We purposively selected the Pokot community where female circumcision remains highly cherished backed by strong cultural systems [5, 18]. Female circumcision among the Pokot is also backed by strong patriarchal norms making life of those not circumcised vulnerable [5]. The Pokot depend on livestock as their main source of livelihood and dowry [3]. Wealth is also an important factor for circumcised women because they fetch more dowry (cows) than their non-circumcised counterparts [46, 50].

We conducted thirty [30] serial interviews with 15 uncircumcised women. We conducted the serial interviews within an interval of one month using the same questions to achieve saturation. Five (05) Key Informant Interviews were conducted with District officials, human rights activist, religious and cultural leaders, who were purposively selected. The sample size for uncircumcised women was determined using Creswell’s (1998) qualitative sampling framework and they were recruited using Respondent Driven Sampling (RDS) technique [47]. A Focus Group Discussion with women irrespective of their circumcision status was organized as an entry point with support of officials from two Non-Governmental Organizations operating in the area (National Association of Women Organization in Uganda and ZOA-Uganda) and Community Development Officers (CDOs) from Amudat District Local Government. The FGD helped us to get initial participants that were later used to locate other study respondents (uncircumcised women).

### Data collection

We adopted Serial Interviewing to collect data from uncircumcised women [48]. Serial Interviewing helped us in verifying and cross-checking the information since some of the participants were reluctant to share their deep experiences in the first interview [48]. After the first interview, details of participants were picked that helped us in tracing them for the second interview after one month. Each Serial Interview took between 40 and 60 min. The Serial Interviews were conducted in the Pokot language, with support of two native female Research Assistants and at places selected by the participants. The Research Assistants (RAs) fluent in Pokot, Swahili and English were trained for two days. During the training, the RAs were retooled on the Interview Guide and Code of Ethics for Research in the Social and Behavioral Sciences involving human participants. Consent of participants to take part in the study as well as having the interviews recorded was also emphasized during training.

An interview Guide with open-ended questions was used during Interviews. Participants were asked to share their understanding of female circumcision, knowledge on procedure and seasons when the practice is performed. Study participants were also asked to describe their own experiences because of their circumcision refusal. There was a lot of flexibility during interviews to allow participants freely express themselves. The interviews with uncircumcised women were conducted in Pokot and fully recorded. A debriefing meeting was organized with Research Assistants (RAs) every day to reflect on the emerging issues and information from the research.

### Data analysis

We adopted Braun and Clarke’s Thematic Framework of analysis [49]. The first step of analysis involved transcribing the recorded audios and translating the transcriptions. The transcriptions were proofread by three independent researchers. Verification of the translations was performed by a Pokot native who was also well-versed with English language from the Institute of Languages-Makerere University. After confirming the translations, we started identifying meaning units, which we labeled with codes. The emerging codes were used to generate categories. Thereafter, we used the categories to generate sub-themes and themes. However, focus was placed on sub-themes and themes mentioned more than two participants. The emerging themes were independently reviewed by three researchers for validity and accuracy. The process of data analysis was highly iterative involving discussing the generated themes back and forth to ensure accuracy and consistency.

### Ethical considerations

The study was approved by Makerere University School of Social Sciences Research and Ethics Committee (MAKSS), registration number REC09.21.493. The study was also approved by Uganda National Council for Science and Technology (UNCST), registration number SS1046ES. Permission to conduct research was also obtained from Amudat District authorities. Since all the study participants were of consent age (above18 years), written informed consent was obtained from them. However, in case of illiterate participants, thumbprints were obtained. The use of thumbprints as a way of obtaining consent was approved by the Research Ethics Committee (REC). Prior to securing informed consent, the Ras abreast the study participants about their inclusion criteria, and the probable risks and benefits associated with the study in their local language (Pokot).

Native female Research Assistants were recruited to carry out interviews with uncircumcised women to reduce emotional and psychological harm. Codes were used during data analysis and report writing to ensure anonymity. Participants were also assured of their freedom of not responding to any questions they didn’t feel comfortable with, and asking for breaks at any time during the interviews without fear of retribution from the study team. Given the fact that interviews were conducted during the COVID-19 pandemic, anti-COVID 19 guidelines and Standard Operating Procedures (SOPs) by the government of the Republic of Uganda were highly observed. The participants were given facemasks and sanitizers as well as encouraged to social distance during interviews.

## Results

### Description of study participants

The findings are based on 30 Serial Interviews with fifteen [15] uncircumcised Pokot women and five [5] Key Informant Interviews from Amudat District. Ten [10] of the participants had attained primary education, while five [5] had secondary education. Nine [9] of the participants were married, four [4] were unmarried while two [2] were still in school. None of the participants were formally employed. Out of the 15 participants, ten [10] were aged 18–25, three [1] aged 26–35 and two [2] aged 35 and above. Table 1, below, is illustrative of the demographic characteristics of participants.

**Table 1:** Profile summary of the participants

Participants	Age	Level of education	Employment Status	Marital status	Location	Religion
P1	37	Secondary	Business	Married	Semi-urban	Protestant
P2	19	Primary	Unemployed	Single	Rural	Pentecostal
P3	21	Primary	Unemployed	Single	Rural	Protestant
P4	22	Primary	Unemployed	Married	Rural	Protestant
P5	31	Primary	Unemployed	Divorced	Rural	Protestant
P6	34	Primary	Unemployed	Married	Rural	Pentecostal
P7	24	Primary	Business	Married	Semi-urban	Pentecostal
P8	27	Primary	Unemployed	Divorced	Rural	Catholic
P9	23	Secondary	Unemployed	Married	Rural	Protestant
P10	21	Secondary	Unemployed	Married	Rural	Protestant
P11	18	Primary	Student	In-school	Rural	Catholic
P12	19	Secondary	Student	In-school	Rural	Protestant
P13	21	Secondary	Unemployed	Married	Rural	Protestant
P14	20	Primary	Unemployed	Married	Rural	Pentecostal
P15	36	Primary	Unemployed	Married	Rural	Protestant

The themes in form of negative experiences of uncircumcised Pokot women that emerged during data analysis were; denial to participate in community and cultural functions, difficulties in getting Pokot marriage partners, denial of conjugal rights (sex) and basic needs, refusal to give names to their children, rejection by elders and relatives, and stigmatization of their children. Three positive experiences also surfaced during data analysis including; sexual enjoyment during sexual intercourse epitomized by ease of reaching orgasms and less complication while giving birth as well as reduced risk of exposure to Sexually Transmitted Diseases (STDs).

### Knowledge about female circumcision

Before recounting their experiences, we asked participants to share their understanding of female circumcision. We also engaged participants to establish their knowledge levels regarding female circumcision procedure. All the participants demonstrated understanding of female circumcision and the different seasons when the practice is performed. Participants described female circumcision as the *“cutting of the female private parts, especially the clitoris using a sharp knife or razor blade”*. The participants further mentioned that female circumcision is performed during the months of March, April, August and September of every even year targeting girls between the age of 10 to 15 years with the intention of preparing them for marriage and ushering them into womanhood.

All the participants stated that a traditional dance “*Naleyo Dance*” is performed in the community to alert the young girls about to undergo circumcision. The participants also reported that the “*Naleyo Dance*” is used to alert relatives to prepare the local brew *“kumi-ket*”, which is drunk by the elders during the circumcision. One of the key informants revealed that on the day of circumcision, the girls are taken to the river to bath and their fathers smear them with black mud on the face as the cutter “*Koko-Melkong”* prepares the stones where the girls sit before, they are circumcised. After circumcision, the girls are baptized *“Chemeril”* and are isolated from the community until when they heal. After healing, the newly circumcised girls are ushered in a dancing procession to the nearby trading center to get gifts *“Meseren”* from the community members.

Besides participants demonstrating their understanding of female circumcision, they also revealed their personal negative experiences associated with their rejection of female circumcision including; denial to participate in community and cultural functions, difficulties in getting a marriage partner, refusal by elders and clan leaders to bless their marriage, denial of conjugal rights and basic needs, and refusal to give names to children of uncircumcised women by husbands and relatives.

### Denial to participate in community and cultural events/functions

Participants narrated how they were denied mixing with circumcised women on several occasions during community meetings on grounds that, they had unpleasant smell. Additionally, uncircumcised women were also barred from contributing ideas during the community meetings. These experiences were augmented by key informants who revealed that, uncircumcised women are denied leadership positions. This is because matters regarding the choice of candidates to be fronted for pokot leadership positions are entrusted with the leaders. As a result, participants expressed feelings of rejection and compromised self-efficacy to co-exist during public gatherings and contest for leadership positions thus alienating themselves from participating in community meetings. This is indicated in the voices of participants below:

*I remember one time I went for a community meeting to discuss about cattle raids in the area but, the elders said that those circumcised should not mix with those not circumcised. I was not even allowed to say something in that meeting. I felt so bad because our animals had also been raided by warriors and I wanted to tell the elders (P1).*

*Uncircumcised women are not allowed to say anything during community meeting because they are looked at as young girls* “*Chepto*”. *Of course, this has forced many young girls to succumb to pressure and get circumcised. My mother is a non-Pokot (muganda) but she was compelled to be circumcised because of what she was going through in the community (Female key informant).*

Besides, community meetings, participants were also not allowed to participate in cultural ceremonies like *“Naleyo”*. Uncircumcised women are also not allowed to serve elders during traditional ceremonies on grounds of constructed myths. For instance, the myth that, uncircumcised women have unpleasant smell and are not yet grown-up women. We recite a story from one of the participants below:

*My cousin sister was getting married and she asked me to serve elders from the husband’s side but one of my uncles who knew that, I am not circumcised stopped me from serving them claiming that I was not clean to serve elders. He also claimed that, I will bring bad blessings to their daughter’s marriage. I felt so sad and decided to stop attending traditional and family functions (P6).*

Participants further highlighted that they continue to be denied access to some public places like water collection points, and kraal to collect cow dung to beautify their houses. Participants reported that this is due to the perception among the elders that, uncircumcised women leak from their private parts. This discrimination is also extended to their children who are always harassed by their peers at the water collection points, and in extreme cases not allowed to fetch water. This is evident in the excerpts below:

*“I remember when I was still young, my stepmother used to say that, I will not get married unless I cut my long and stinking clitoris between my legs. Life was hard because one time she stopped me from going to the granary, grinding maize, and milking cows at home claiming that, my long and stinking clitoris will bring curse and food insecurity to the family”. (P15)*

*“Sometimes when we go to the water collection points, those circumcised women tell us to first wait for them to fill their jerricans before we make the water dirty since we leak from our private parts.” (P12)*

### Difficulties in getting a marriage partner

Both married and unmarried participants mentioned difficulties in getting a pokot marriage partner. Participants attributed this to the myth that uncircumcised women are a curse to the family. This was exacerbated by hilarious names given to uncircumcised women like “*Chemalayinan”* and *“Chineyomegtilinye”* literally meaning a prostitute and dirty woman respectively. As a result, Pokot men shun away from falling in love with uncircumcised women for fear of insults and bullying from the community members. Some of the participants revealed having abandoned their original communities to other areas where their circumcision status was not known to get marriage partners. For example, one participant stated,

*Boys in the village used to tell me that, I will not get someone to me marry because I am not circumcised. I was forced to leave the village and come to Amudat Town Council, where nobody knows that I am not circumcised and got a boyfriend (P7)*

### Refusal by elders and clan leaders to bless their marriage

Participants revealed that marriage blessings in their culture are given by elders and clan leaders. However, these blessings have been tied to female circumcision. Participants further revealed that uncircumcised women are always blamed for their marriage misfortunates. Besides, elders and clan leaders are not supposed to visit their homes. This is indicated in the statements below made by some participants:

*I remember one time my father-in-law insulting me in front of my children by saying that look at this girl with seven horns. You know the shaper end of the clitoris looks like a horn. I felt embarrassed and degraded in front of my children and decided to leave that home (P9).*

*I am always harassed and abused by my in-laws whenever I try to go and pick food from the granary, claiming that, I will cause food insecurity to the family. I have tried several times to tell my husband to secure a plot of land far from his parents, but he is always influenced by his parents. The harassment I am going through in this home is too much (P15).*

The participants further highlighted that, the economic benefits in form of dowry tagged on circumcised women during marriage ceremonies have led to elders and clan leaders not blessing the marriages of uncircumcised women. The elders’ economic expectations in form of dowry from the marriages of uncircumcised women are so low. Culturally, the Pokot elders are believed to be the custodians of blessings. The uncircumcised women revealed that their parents are paid less or no dowry at all compared to their circumcised counterparts. This is evident in the excerpt below:

*What disturbs me so much is that, the parents of my husband refused him from paying dowry (cows) to my parents because I am not circumcised. They even vowed never to come and visit us at my home. My children are always discriminated and stigmatized against whenever they go to visit their paternal grandparents. They do this to my children because I refused to undergo circumcision (P6).*

### Denial of conjugal rights and basic needs at home

Married participants revealed that their husband denied them conjugal rights (sex) and other basic needs after realizing that they were not circumcised. This was done to pressure them to undergo female circumcision. The pressure to circumcise has been exacerbated by their co-wives who influence their husbands not to have sex with them claiming that, dirty things come out of their vagina, have unpleasant smell and engage in extramarital affairs. Two participants revealed they had deserted their matrimonial homes due to pressure from their co-wives. Other participants reported engaging in physical fighting with their husbands and co-wives over allegations of extramarital affairs. Two participants had this to say in light of these physical fights:

*I remember one time my husband got a full cabbage, boiled it and placed it on the table. When I asked him how he was going to eat the full cabbage that is not cut, he told me that, I am like that cabbage. That I first go for circumcision and come back when I am like ready cabbage to have sex with me. I felt so bad and decided to leave him and started staying with my mother. I don’t feel like reconciling with that man because he mistreated and starved me sexually (P8).*

*My husband got another woman after telling him that I am not circumcised. One time that woman confronted me in front of my children saying that, she is the only woman in the family who doesn’t smell. My friend, I grabbed her and by the time people came to separate us, I had already given her enough ahahaha (P6).*

### Refusal to give names to children of uncircumcised women by husbands and relatives

Married participants mentioned that their husbands with support of relatives, after discovering that, they were not circumcised refused to give surnames to their children claiming that, the children are a “bad omen” to the family. Participants further expressed that, derogatory and humorous names like *“magangandet”* literally meaning outcast are given to their children to depict them as a curse to the family. The respondents expressed frustration over the deliberate decision by their partners’/relatives’ refusal to name their children. Though these women were not willing to be circumcised, they cherished the naming of children as they cited some of the advantages associated with the rite; it was necessary for determining someone’s belonging in terms of the family lineage and clan. The Pokot surnames also have inherent meaning; usually reflecting someone’s birth-story. This is evident in the excerpt below:

*In our culture, the husband or father-in-law is the one to name after the child because that name is used as a clan or family identity and sometimes help to talk about the child’s birth story. I struggled with my husband to give names to our children because his parents and elders had asked him not to name them saying that, they were a curse to the family (P6).*

The participants further stated that, discrimination and stigmatization in form of giving derogatory names to their children have been extended in schools forcing some of their children to drop out of school. Participants attributed this to persistent bullying and teasing by their peers. We present this in three narratives from two participants:

*My children are not allowed to socialize or interact with other children in the community, especially neighbors because they are considered outcasts and unclean. They are always called all sorts of names and mocked by their peers because of my position against female circumcision (P4).*

*My son was forced to drop out of school because of the persistent teasing and bullying from peers that, he is a son of a woman who is not clean. I reported several times to the school administrators for help but I was not helped until when my son dropped out of school. What annoyed me so much is that, even my husband did not help our child to be resettled in another school (P15).*

*One day I sent my child to the neighborhood to collect cow dung for beautifying our house but the man chased him and called him all sorts of derogatory names because I am not circumcised. He shouted at him saying he should not collect cow dung from their kraal because he will bring a curse on their family. They have also stopped my children from socializing with their children (P4).*

### Enjoying sexual intercourse

Participants mentioned that enjoying playing sexual intercourse with their husbands and boyfriends was one of the positive experiences. Women who refused to be circumcised reported experiencing high sexual sensation and satisfaction during intercourse with their partners. Participants revealed having received several complaints from their friends who were circumcised that, they feel a lot of pain while having sexual intercourse. This is evident below:

*I have my friend who is circumcised but she tells me that she lost interest in playing sex with her husband because she feels a lot of pain whenever they have sex and sometimes bleeds. Personally, I have never felt pain apart from the very first time I had sex because I was still a virgin (P4).*

*I feel happy whenever I have sex with my husband because I enjoy it a lot unlike circumcised women whose husbands report feeling a lot of pain on vaginal penetration because the vulva (vaginal opening) area is very small (P6).*

### Less complications while giving birth

The participants who had given birth reported less complications while giving birth as one of the positive experiences of not being circumcised. This is evident in the excerpt below:

*I have five children so far, and I have never got any problem while giving birth yet my friends who are circumcised have delivered from Moroto Hospital. Some of my friends have undergone caesarean section after failing to give birth from our lower-level health facilities. These experiences have taught me a lesson and I pledged not to circumcise my daughters although my relatives sometimes try to influence me to circumcise my daughter (s) (P6).*

### Reduced risk of exposure to sexually transmitted diseases

Participants mentioned that, their refusal to undergo female circumcision reduced their risk of exposure to sexually transmitted diseases. Participants reported that, the same ‘‘*wembe’’* or knife was used to circumcise many girls at the same time, which exposes them to sexually transmitted diseases. These revelations are evident below;

*‘I remember female circumcision was very prevalent among the Pokot at the time when HIV/AIDS was also at the peak in Uganda and most people were not even aware of the virus. Circumcisers were using the same knife on more than one girl and some girls in the community got infected. I am very sure people like me survived getting infected because of refusal to undergo female circumcision. (P7)*

The above revelation by an uncircumcised woman were reaffirmed by one of the health officials from Amudat District who noted,

*‘The ‘‘wembe’’ that is used for cutting usually transmits diseases to girls since it’s used to cut multiple girls at the same time’’. Of course, many of these girls could not know that they had been exposed to such diseases because Pokot was a closed community with limited access to social services like health and education (Female key informant)*

## Discussion

In this paper, we have examined the lived experiences of uncircumcised Pokot women in North-Eastern Uganda. Whilst the prevalence of female circumcision at the national level is 1.4%, the practice remains high (54%) among the Pokot community [17, 18, 53]. The findings provide evidence that, uncircumcised Pokot women face a plethora of psychosocial challenges, as they continue to live in a community, where the practice is cherished. The act of denying uncircumcised women mixing with circumcised women during community meetings and engaging in cultural events was identified as one of the terrible experiences. Consequently, many uncircumcised women have been denied participation in decision-making processes as noted by previous scholars [19, 23, 51, 52]. Whilst the Constitution of the Republic of Uganda, the Prohibition of FGM Act, Succession Act, Equal Opportunities Commission Act, and Domestic Violence Act are very pronounced on the rights of women, cultural myths continue to dominate the Pokot community. Uncircumcised Pokot women are constantly refused from engaging in decision-making foras on claims that, they are still girls *“chepto”*. Awich [46], noted that, the denial of uncircumcised women from filling leadership positions has been worsened by the patriarchal system of social organization. The system advances women dependence on men and elders in the community as opposed to self-sufficiency.

The myth among the Pokot community that an uncircumcised woman remains a girl until she goes through the ritual continues to be exploited by elders to extend their preeminence and control over resources [41, 53, 54]. Notwithstanding, uncircumcised women, especially from urban centers, and those with some literacy levels have challenged the odds by contesting in high political offices. Therefore, education as noted by previous scholars remain a key factor in influencing power relations and control over resources among the female circumcision communities [13, 24, 54, 55].

This study has revealed that, uncircumcised women experience difficulty in getting marriage partners. This was partly attributed to the hilarious names given to uncircumcised women like “*Chineyomegtilinye,”* literally meaning dirty woman, thus scaring away potential intimate partners. As a result, some of the uncircumcised women who cannot stand the humiliation, relocate to areas where their circumcision status is not known to get intimate partners. This is consistent with previous studies which observed that cross-marriage is key in building cultural competence and co-existence [56, 57]. Besides, difficulty getting a pokot marriage partner, the bride price attached to circumcised women has made uncircumcised women to undergo unbearable experiences. This, too, is consistent with previous studies [18, 54].

As this study has revealed, uncircumcised women encounter multiple marital related challenges; including, failure to name their children by their husbands and relatives, denial of conjugal rights and basic needs at home and refusal to bless their marriage. The situation is exacerbated by the actions of circumcised co-wives who always tell their husbands not to have sexual intercourse with uncircumcised co-wives claiming that, they are unclean, smell badly and engage in extramarital affairs. These myths have contributed to intimate partner violence and child abuse. Nevertheless, these sexual myths have been disapproved [19, 60, 61]. The sexualisation of the woman’s body and polygamous marriage system among female circumcision communities have also worsened matrimonial challenges among the uncircumcised women [17, 19, 23, 53, 59, 62–65]. The situation has been compounded by the inadequate psychosocial support towards uncircumcised. As a result, uncircumcised women desert their conjugal homes and in worse situations develop suicidal and murder ideations/thoughts. An interaction with key informants revealed that, several uncircumcised married women have divorced due to persistent harassment by their husbands and relatives. These findings conform with previous scholars who discovered that, matrimonial challenges that come with circumcision refusal are not bearable [6, 18, 20, 25, 66–68].

Most participants noted that, discrimination and stigmatization that come with circumcision refusal is extended to their children in the community and in schools. As a result, some of their children are forced to drop out of school due to persistent teasing and bullying from their peers. Similar reactions characterized by ostracism and stigmatization towards children of uncircumcised women were re-echoed by key informants at the district level. Some of the participants revealed that, efforts to get redress from school administrators regarding their children at school have proved futile. These barbaric behaviours against uncircumcised women and their children have been reaffirmed by previous authors on the effects of deviant behaviours in the community [38, 66, 67].

Although the promoters of female circumcision have subjected uncircumcised women to unbearable experiences as mentioned above, participants revealed some positive experiences associated with the refusal to circumcise. Evidence has been documented on the relationship between female circumcision and birth complications. Several medical experts concur that, uncircumcised women are likely to suffer less complications while giving birth as compared to their counterparts [25, 55, 58, 61, 68, 69]. This was reaffirmed by participants who reported receiving less complication while giving birth as compared to their counterparts who were circumcised.

The participants further reported that, female circumcision exposes those undergoing the ritual to STDs since the same cutting tool is used on all the girls. Participants also reported reduced exposure of uncircumcised women to HIV/AIDS during sexual intercourse. This they attributed to the fact that they private parts are intact and require little friction during sexual intercourse. However, these revelations without any scientific evidence are not only likely to escalate the spread of STDs, but also undermine the campaigns of family planning. This confirms findings by previous scholars that, uncircumcised women were less likely to use contraceptives as compared to circumcised women [70].

The study also revealed improved sexual functioning (high sexual pleasure) as one of the key positive experiences. Even though little evidence is available to support this argument, medical professionals have confirmed that, tampering with the woman’s clitoris and labia minora may result into development of inelastic scars and adhesions around the excised areas thus causing impaired sexual functioning [6]. Earlier studies on female circumcision picked voices of feeling pain during sexual intercourse by circumcised women [61]. Our study findings point to the importance of overcoming stigmatization of uncircumcised women to enable them share their positive experiences/benefits of being uncircumcised. This will boost campaigns against female circumcision in practicing communities.

Several scholars concur that, female circumcision is a deeply entrenched cultural practice among the Pokot community in north eastern Uganda. The practice is also perceived as the only way of ushering girls into adulthood [2, 40, 46]. Legal instruments have been developed against the practice; however cases of female circumcision continue to be reported due to strong social values and psychosocial distress experienced by those not circumcised [72, 73]. Therefore, documenting the positive experiences associated with not being circumcised can be a blessing in the fight against the practice.

## Conclusion

Uncircumcised women have been exposed to a plethora of unbearable experiences making co-existence in the community difficult. Denial of participation in community and cultural functions, rejection by elders and relatives, difficulties in getting marriage partners, denial of conjugal rights and basic needs, and refusal to give names to their children. Therefore, developing integrated psychosocial support programmes involving all stakeholders and documentation of scientifically proven benefits can be exploited as a beacon of hope to navigate the negative experiences because of circumcision refusal. Notwithstanding, this study’s findings are based on a small convenience sample drawn from a single community yet female circumcision is practiced in more than five communities. Thus, generalization of the study results to other communities without similar contextual factors is limited. Further studies need to be conducted in other communities practicing female circumcision.

## Data Availability

The datasets/transcripts used and analyzed for the study are available. from the corresponding author on reasonable request.

## Abbreviations

CDO: Community Development Officer.
FM: Female Circumcision.
FGM: Female Genital Mutilation.
RDS: Respondent Driven Sampling.
UDHS: Uganda Demographic and Health Survey.
UWEP: Uganda Women Entrepreneurship Programme.
MAKSSREC: Makerere University School of Social Sciences Research and Ethics Committee.
UNCST: Uganda National Council for Science and Technology.
